# Down-Regulation of Integrin β1 and Focal Adhesion Kinase in Renal Glomeruli under Various Hemodynamic Conditions

**DOI:** 10.1371/journal.pone.0094212

**Published:** 2014-04-04

**Authors:** Xiaoli Yuan, Wei Wang, Juan Wang, Xiaohui Yin, Xiaoyue Zhai, Lining Wang, Kai Li, Zilong Li

**Affiliations:** 1 Department of Nephrology, First Affiliated Hospital of China Medical University, Shenyang, China; 2 Department of Histology and Embryology, Institute of Pathology and Pathophysiology, China Medical University, Shenyang, China; 3 Department of Surgical Oncology, First Affiliated Hospital of China Medical University, Shenyang, China; 4 Department of Nephrology, General Hospital of Tisco, Shanxi Medical University, Taiyuan, China; Fondazione IRCCS Ospedale Maggiore Policlinico & Fondazione D'Amico per la Ricerca sulle Malattie Renali, Italy

## Abstract

Given that integrin β1 is an important component of the connection to maintain glomerular structural integrity, by binding with multiple extracellular matrix proteins and mediating intracellular signaling. Focal adhesion kinase (FAK) is the most essential intracellular integrator in the integrin β1-FAK signalling pathway. Here, we investigated the changes of the two molecules and visualized the possbile interaction between them under various hemodynamic conditions in podocytes. Mice kidney tissues were prepared using in vivo cryotechnique (IVCT) and then were stained and observed using light microscopy, confocal laser scanning microscopy and immunoelectron microscopy. The expression of these molecules were examined by western blot. Under the normal condition, integrin β1 stained continually and evenly at the membrane, and FAK was located in the cytoplasm and nuclei of the podocytes. There were significant colocalized plaques of two molecules. But under acute hypertensive and cardiac arrest conditions, integrin β1 decreased and stained intermittently. Similarly, FAK decreased and appeared uneven. Additionally, FAK translocated to the nuclei of the podocytes. As a result, the colocalization of integrin β1 and FAK reduced obviously under these conditions. Western blot assay showed a consistent result with the immunostaining. Collectively, the abnormal redistribution and decreased expressions of integrin β1 and FAK are important molecular events in regulating the functions of podocytes under abnormal hemodynamic conditions. IVCT could offer considerable advantages for morphological analysis when researching renal diseases.

## Introduction

Glomerular podocytes are terminally differentiated cells that line the outer aspect of the glomerular basement membrane (GBM). The GBM forms the final barrier against protein loss, which explains why its dysfunction causes protein leakage into the urine, resulting in proteinuria [1]. Podocytes are injured in many types of human and experimental glomerular diseases, including hypertensive renal disease [2]–[4].

As an important component of the connection between podocytes and the GBM, the reduction of integrin α3β1 might represent one of the mechanisms of podocyturia in glomerular disease. Integrin α3β1 is a membrane glycoprotein consisting of two subunits, a larger α chain and a smaller β chain [5], and the alteration of subunit β1 can represents the change of integrinα3β1. Under different stimuli, integrin α3β1 binds to multiple extracellular matrix (ECM) proteins, including laminin, collagen and fibronectin, which are the components of GBM and which transduce different intracellular signals [6]. At binding sites, they form focal contacts, which bring together cytoskeletal and signaling proteins during the processes of cell adhesion, spreading and migration. In many intracellular “integrator”, FAK is the most essential one, which is a nonreceptor protein tyrosine kinase. FAK appears to play a major role in mediating signals. Phosphorylation at Tyr397 might be the first step or an indispensable path in further signaling transduction. It has been demonstrated that FAK activation is significantly increased after podocyte injury. More recent studies have shown that inhibiting FAK activation reduces proteinuria and podocyte effacement [7]. It has been demonstrated in many experimental animals that FAK and FAK phosphorylation are increased in many pathological situations and that they are translocated in the nucleus [8], [9], but the results was obtained in some chronic diseases or for a long period after administering treatment in vitro. Nevertheless, how these molecules change in the acute diseases or during the prophase of abnormal hemodynamic conditions remains to be examined.

It is well known that hemodynamic factors, such as blood flow and pressure, exert an important influence on the native structure and function [10]–[12], Acute hypertension, high pressure of glomerular blood capillaries impaired the size-selective barrier function of the slit diaphragm and glomerular basement mambrane, so that hyperfiltrated serum proteins are processed [13], [14]. Cardiac arrest condition, ischemia and hypoxia, was reported to induce some changes in glomerular structures and also damages of renal microvascular cell-cell junctions, which then increased vascular permeability and local interstitial edema [15], [16]. These influences appear in a split second, so we must maintain all of the components in situ to study the actual situation, which is impossible to achieve if we prepare the organs using conventional methods [17]. In contrast, IVCT is a technology that can arrest transiently dynamic structures in living animal organs. Moreover, IVCT has been used successfully to clarify the serum protein passing through the glomerular capillary loops under various hemodynamic conditions [18]. All of these experiments have indicated that IVCT is a sensitive and powerful fixation technique for visualizing the structural and functional changes that occur in living animal kidneys under various hemodynamic conditions.

In the present study, the alterations of integrin β1 and FAK in mouse kidneys under various hemodynamic conditions were visualized using IVCT in combination with freeze-substitution, and they were further analysed quantitatively by western blot. Our findings provide new insight into the mechanism of proteinuria during hemodynamic disorders.

## Materials and Methods

### 1. Antibodies

Rabbit monoclonal (EP695Y) against FAK, mouse monoclonal (P5D2) against integrin β1, and donkey anti-rabbit IgG/TRITC and goat anti-mouse IgG/FITC antibodies were obtained from Abcam (Cambridge, MA, USA). Rabbit polyclonal against FAK phosphotyrosine at 397, biotinylated sheep anti-rabbit IgG and biotinylated rabbit anti-mouse IgG antibodies were purchased from Sigma (St. Louis, MO, USA).

### 2. Animals

Adult C57BL/6J mice, weighing 20–30 g, were used. The experimental animal procedures were approved by the Animal Experimental Committee of China Medical University.

### 3. Preparation of kidney tissues

#### 3.1. IVCT for Mouse Kidneys, Freeze-Substitution Fixation and Paraffin-Embedding

The mice were classified into three groups, each containing five mice: a normotensive group; an acute hypertensive group; and a cardiac arrest group. The mice were anesthetized via intraperitoneal injection of sodium pentobarbital (50 mg/kg body weight). In the normotensive group, we resected the left kidney under normal blood circulation. In the acute hypertensive group, we prepared an animal model by ligating the abdominal aorta just below the branching renal arteries for 10 min [19], before removing the left kidney. The cardiac arrest animal model was achieved by injecting excessive amounts of the anesthetic and fixing the left kidney immediately after the heart stopped beating.

The renal tissues were then assessed with IVCT, which has been described in previous reports [10], [14], [19]. Briefly, a cryoknife precooled in liquid nitrogen (−196°C) was positioned over the left kidney of the mouse. The kidney was immediately cut with the cryoknife, and liquid isopentane-propane cryogen (−193°C) was simultaneously poured over it. The frozen kidneys were carefully trimmed with a dental electrical drill in liquid nitrogen. Some of the frozen specimens were transferred to freeze-substitution, as described below, while others were preserved in liquid nitrogen for biochemical examination.

The pieces were freeze-substituted in absolute acetone containing 2% paraformaldehyde (PFA) cooled in dry ice-acetone at −80°C for 48 h and then were gradually rewarmed to room temperature. They were washed in pure acetone twice, transferred into xylene, and then embedded in paraffin wax.

#### 3.2. Immerse-Fixation of Resected Kidney Tissues

As a control group, five kidney specimens were routinely treated with conventional fixation procedures. Some pieces of the tissues were immersed in 4%PFA for 24 h at room temperature, followed by gradual dehydration in a series of alcohols and then transfer into xylene. Finally, the samples were embedded in paraffin wax. The remainder was maintained in −80°C for the biochemical examination.

### 4. Immunostaining on Deparaffinized Sections

The paraffin-embedded tissues were cut at a 5 μm thickness and were deparaffinized with xylene and a graded series of alcohols. Some of the sections were stained in common hematoxylin and eosin (HE) to observe them morphologically. Other sections were incubated with 3% hydrogen peroxidase (H_2_O_2_) to block the non-specific reactivity of endogenous peroxidase, followed by a wash with phosphate-buffered saline (PBS). The samples were repaired with sodium citrate buffer liquid at high pressure and were blocked with PBS, containing 5% normal bovine serum (Boster, Wuhan, China) for 1 h at 37°C. Some sections were subsequently incubated with the primary antibodies at 4°C overnight, Other sections were incubated with PBS without the primary antibody. After washing them three times with PBS, the sections were incubated with corresponding secondary antibodies at 37°C for 1 h. Thereafter, they were incubated with horseradish peroxidase (HRP)-conjugated avidin-biotin complex (ABC) for 20 min and were visualized with metal-enhanced 3′3-diaminobenzidine (DAB) (Boster, Wuhan, China) for 5 min (ABC-DAB method). To visualize a clearer structure, we stained them with hematoxylin for 1 min and then dehydrated them in a graded series of alcohols and xylene. Finally, the specimens were sealed with peucine and were photographed under a light microscope or a confocal laser scanning microscope (FV10-ASW2.1 Viewer). Simultaneously, the integral optical density (IOD) of the target protein was measured with Meta Morph (UIC, US), and the result was determined as the sum of the glomeruli. IOD was defined as the sum of the optical densities of all the positive pixels in the image, which represents the quality of the targeted protein.

### 5. Immunoelectron Microscopy

To detect the ultrastructural changes of glomeruli and the immunolocalization of podocyte proteins under various hemodynamic conditions, we stained some sections with 1% osmium tetroxide acid for 20 min after DAB dyeing, dehydrated them with a graded series of alcohols and acetone, and finally inversion-embedded with Epon 812, polymerized at 60°C for 48 h. The sections were cut at a 70 nm thickness and stained with uranyl acetate, and then ultrastructural images were obtained with a transmission electron microscope.

### 6. Western Blot Analysis

The glomeruli were isolated as previously described [19]. Briefly, cortical kidney tissue was finely minced and sieved through sequential sieves of 250, 150, ana 105 μm pore size. The purity of the glomeruli obtained was 92% to 96% with minor tubular contamination. We performed western blot with the enhanced chemiluminescence system. We prepared two equal amounts of glomeruli. One was homogenized in buffer (10 mmol/L Tris-HCI, 50 mmol/L NaCl, 5 mmol/L EDTA, and 1% TritonX-100, containing phosphatase and proteinase inhibitors) to obtain the total proteins (TPs). It was maintained at 4°C overnight. The other was homogenized in buffer (10 mmol/L HEPES, 10 mmol/L KCI, 0.1 mmol/L EDTA, 0.1 mmol/L EGTA, and 1 mmol/L DTT, containing phosphatase and proteinase inhibitors) to obtain cytoplasmic proteins (CPs) and nuclear proteins (NPs). Then, all of the homogenates were centrifuged, and the supernatants, which contained cytoplasmic proteins, were collected; the remainder was lysed again at 4°C overnight with the same buffer that was used in total protein extraction.

Proteins were electrophoresed on 10% SDS-polyacrylamide gels and were electrotransferred onto polyvinylidene difluoride membranes. After blocking with 5% non-fat milk for 1 h at room temperature, the membranes were hybridized with specific primary antibody overnight at 4°C. The filters were then washed with PBS-0.1% Tween 20 and were incubated with corresponding HRP-conjugated secondary antibody for 2 h at room temperature. We detected immunoreactivity with the enhanced chemiluminescence system (Amersham Biosciences, Buckinghamshire, UK). The chemiluminescent signal was captured with a Fujifilm LAS-4000 luminescent image analyzer (Fujifilm, Tokyo, Japan). Equal protein loading was confirmed by GADPH and Lamin B western staining of the gel. The data shown are representative of at least three independent experiments with similar results.

### 7. Statistical Analysis

The values are expressed as the means ±SDs. For multiple comparisons with a single control, one-way analysis of variance (ANOVA), followed by Dunnett's test, was employed. The analyses were conducted using SPSS statistical software, version 17.0. P<0.05 was considered to be a statistically significant difference.

## Results and Discussion

### 1. IVCT Exhibits a Clearer Morphological Alteration of the Glomeruli under Different Hemodynamic Conditions

**Figure 1:**
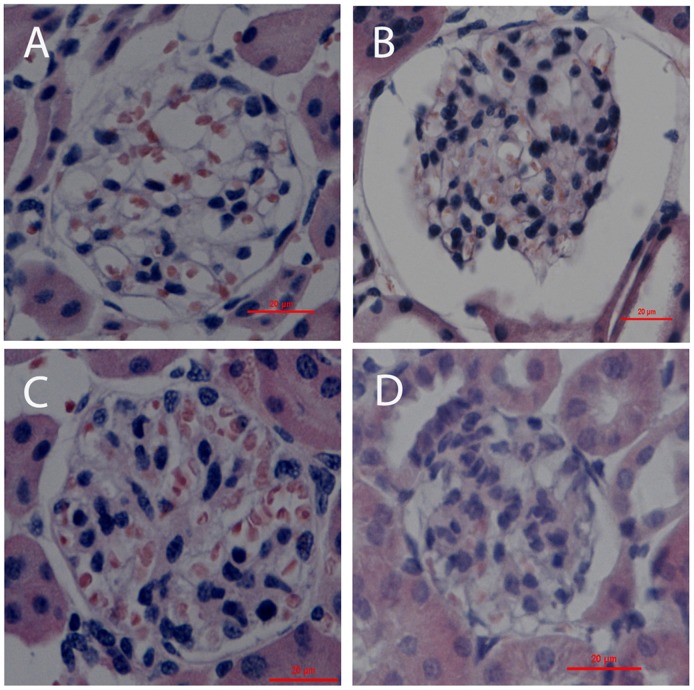
Light micrographs of mouse renal cortical tissues with HE staining. The tissues were prepared by in vivo cryotechnique (IVCT) under normotensive condition (A) and acute hypertensive (B), and cardiac arrest (C) condition, as well as resection kidney tissue processed by immersion-fixation methods under normotensive condition (D). Under the acute hypertensive condition, the Bowman's space and luminal spaces of the proximal and distal tubules were widely open (B), but not in the other two IVCT groups (A,C). Scale bars  = 20 μm.

To examine the native morphology in the mouse kidneys from different groups, we stained the deparaffinized sections with HE. For the sections prepared by IVCT, the capillary loops were smooth and plump under normotensive condition (Fig. 1A). However, they became wizened under acute hypertensive condition, while Bowman's space and the proximal tubules or distal tubules (Fig. 1B) were more widely opened than under normotensive or cardiac arrest conditions (Fig. 1C), as reported before [18], [20]. In all of the above images, there were certain erythrocytes in the blood vessels. In contrast, the capillary loops in sections fixed by immersion were shrunken and there were hardly any erythrocytes in them (Fig. 1D). It was further confirmed quantitatively that the glomeruli from immersion fixation tissues were significantly contracted compared to the IVCT group [17].

Through IVCT, we obtained clearer images of the glomeruli, which visibly showed the instant morphological alterations that occurred under the different hemodynamic conditions.

### 2. Instantaneous Changes in the Distribution and Alteration of Integrin β1, FAK and pTyr-397 FAK under Various Hemodynamic Conditions Are Examined by Immunohistochemistry and Immunofluorescence Analysis

**Figure 2:**
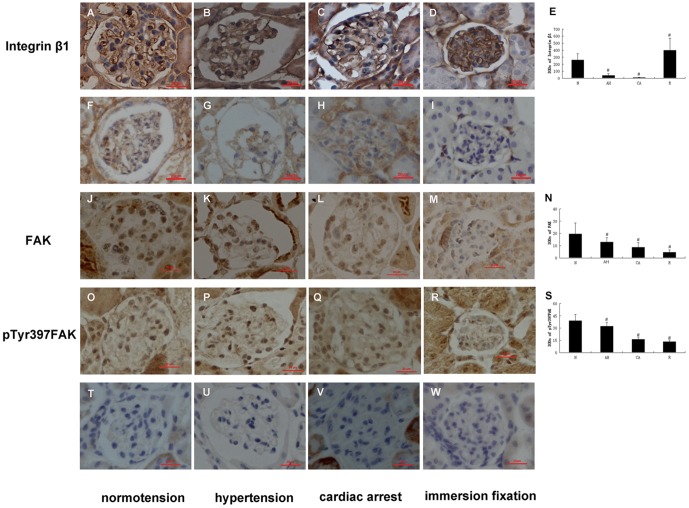
Immunohistochemical localization of podocyte proteins in kidney tissues. The tissues were prepared with IVCT and immersion-fixation methods under various hemodynamic conditions. The micrographs of A, B, C, and D show the localization of integrin β1; F, G, H and I exhibit control stainings ommitting the integrin β1 primary antibody; J, K, L and M show the FAK localization; and O, P, Q, and R present the localization of p-Tyr397 FAK. T, U, V and W show the control stainings ommitting the FAK or p-Tyr397 FAK primary antibody. Immunohistochemical localizations of three proteins prepared with IVCT are shown under normotensive condition (A, J, O), acute hypertensive condition (B, K, P), and cardiac arrest condition (C, L, Q). The tissues in D, M, and R were treated by the immersion-fixation method. Figs. 2E, 2N, and 2S show the IODs of each protein under various hemodynamic conditions. N: normotension; AH: acute hypertension; CA: cardiac arrest; R: resected tissue. Scale bars  = 20 μm. Histogram shows integral optical densities of each protein. IOD: integral optical densities. #: P<0.05.

**Figure 3:**
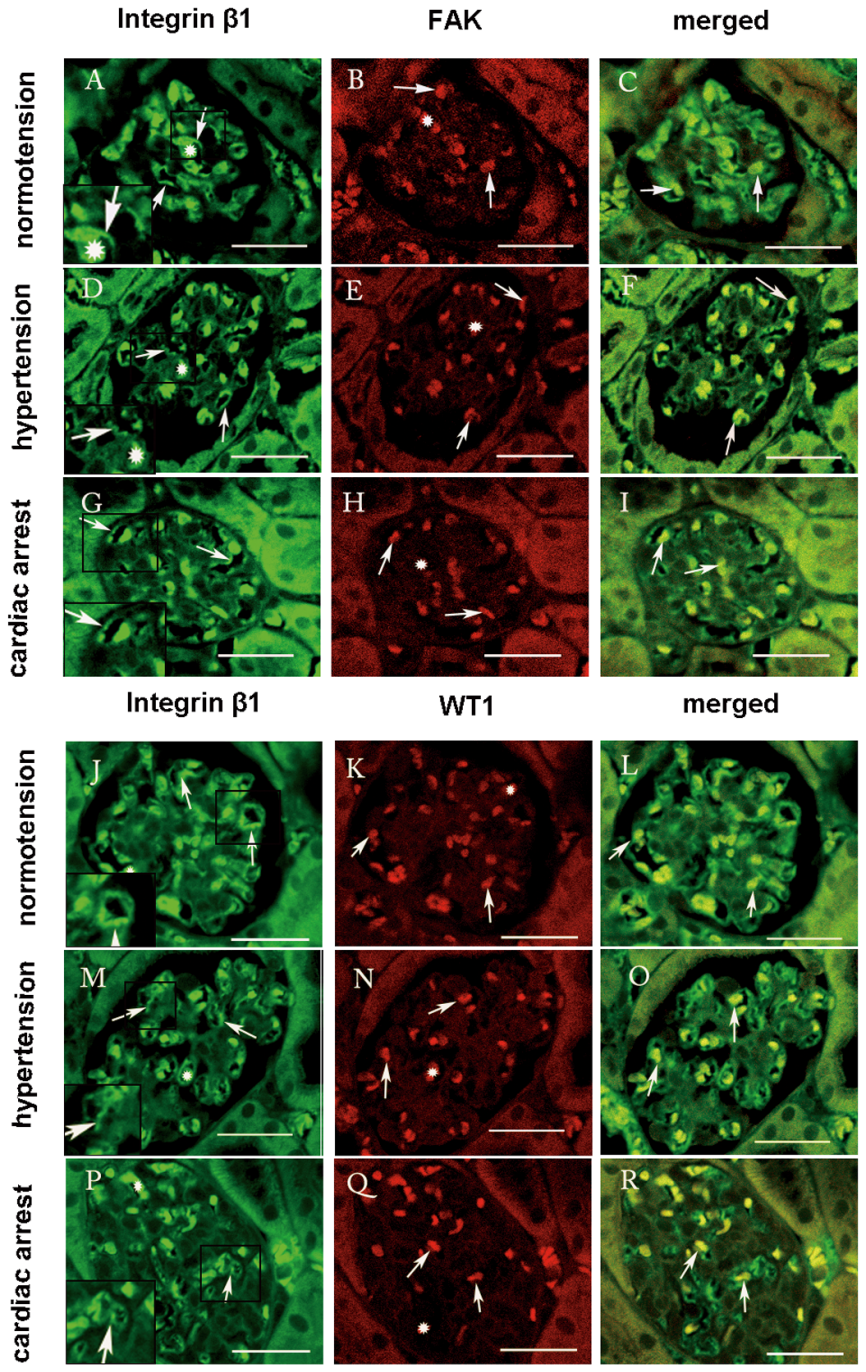
Confocal laser scanning micrographs show the double-fluorescence of integrin β1-FAK (A–I), and integrin β1-WT1 (J–R). Under normotensive condition, integrin β1 showed intense staining along the GBM of the glomeruli (A, arrows). FAK was distributed in the cytoplasm and nuclei of the podocytes evenly (B, arrows). WT1 was localized in the podocytes as well (K, arrows). Under acute hypertensive condition, the immunoreactivity of integrin β1 decreased and stained intermittently (D,arrows), while FAK was restricted to the nuclei of the podocytes (E, arrows), and both of FAK and WT1 decreased. Under the cardiac arrest condition, the immunoreactivity of integrin β1 with FAK and WT1 was obviously reduced (I, R, arrows), compared with the normotensive condition. The double-staining micrographs are shown in C, F, and I (integrin β1 and FAK, arrows), and in L, O, and R (integrin β1 and WT1, arrows). Both of them were distributed in erythrocytes (asterisk). Left bottom pictures are the manified micrographs. Scale bars  = 20 μm.

Immunolabeling showed integrin β1 staining along the glomerular capillary loops (GCLs) continually and evenly under normotensive condition (Fig. 2A), while under acute hypertensive conditions, it decreased and stained intermittently. (Fig. 2B). In the cardiac arrest group, we could only observe weak immunolocalization (Fig. 2C). At the same time, FAK and pTyr-397FAK were distributed in the cytoplasm and nuclei of the podocytes under normotensive condition (Fig. 2J, 2O), but they decreased and became uneven under abnormal hemodynamic conditions (Fig. 2K–2M, 2P–2R). The alterations of integrin β1 and FAK were further confirmed by double immunofluorescent staining (Fig. 3A–3I). Under abnormal hemodynamic conditions, the changes of integrin β1 and FAK were similar with that were observed by immunohistochemical method. In addition, we found the translocation of FAK to nuclei were very obvious under abnormal conditions compared with normal condition. Given integrin β1 and FAK were also immunolocalized in erythrocytes. To provide accurate localizations of two molecules we used WT1, a specific protein of podocytes, as a marker. In Fig. 3J–3R, we performed double immunofluorescence of integrin β1 and WT1. Under abnormal hymodynamic conditions, the colocalization of integrin β1 and WT1 decreased. Unexpectedly, in the immersion-fixation group (Fig. 2D), the integrin β1 staining was the strongest among the four groups. In contrast, the immunolocalization of FAK and pTyr-397FAK was the weakest compared with the IVCT groups (Fig. 2M, 2R). Figs. 2E, 2N, and 2S show the IODs of each protein under various hemodynamic conditions. These results are expressed as the means ±SDs.

### 3. Immunoelectron microscopy shows ultrastructural changes of integrin β1 and FAK expression in the hypertensive condition

**Figure 4:**
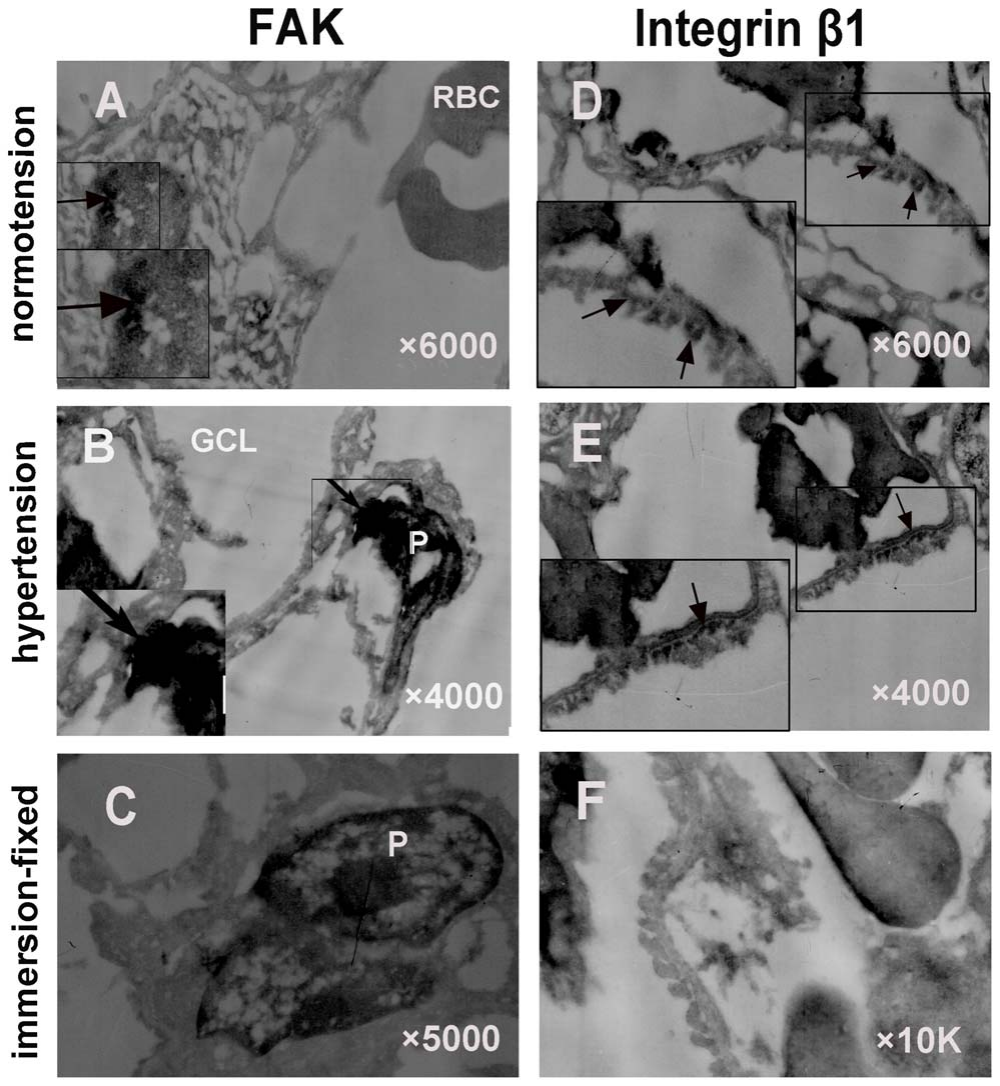
Immune electron micrographs of integrin β1 and FAK in glomeruli under normotensive and acute hypertensive conditions. Under normotensive condition, the foot processes of podocytes were shown to be shrunken with the conventional fixation method (F). In contrast, using IVCT, the foot processes tightly approached each other and became flatter, integrin β1 was distributed on the basolateral membrane of podocytes (D), while FAK was located in the cytoplasm and nuclei of the podocytes (A). In contrast, under hypertensive condition, integrin β1 attached along the basal membrane only (E), while FAK was strikingly gathered in the nuclei (B). Left bottom pictures are the manified micrographs. They were manified 10 K. RBC: red blood cell; GCL; glomerular capillary loop; P: podocyte. (Magnification, ×6000 for A and D; ×4000 for B and E; ×5000 for C; ×10 K for F).

To visualize the topographical migration of integrin β1 and FAK under hypertensive condition, we detected them using immunoelectron microscopy (Fig. 4). In the normotensive group (Fig. 4D), we observed that the foot processes tightly approached each other and became flatter, compared to the immersion fixation group (Fig. 4F). In contrast, the foot processes appeared different degrees of fusion in the hypertensive group (Fig. 4E). Integrin β1 was distributed on the basolateral membrane of podocytes under normotensive conditions (Fig. 4D), whereas FAK was located in the cytoplasm and nuclei of the podocytes (Fig. 4A). In contrast, under hypertensive condition, integrin β1 assembled along the basal membrane only, while FAK was strikingly gathered in the nuclei, indicating the rapid translocation of integrin β1 and FAK during the prophase of the aorta ligation process (Fig. 4B, 4E).

### 4. Acute Hypertensive and Cardiac Arrest Conditions Decrease the Expression of Integrin β1 and FAK and Translocate FAK under Abnormal Hemodynamic Conditions

**Figure 5:**
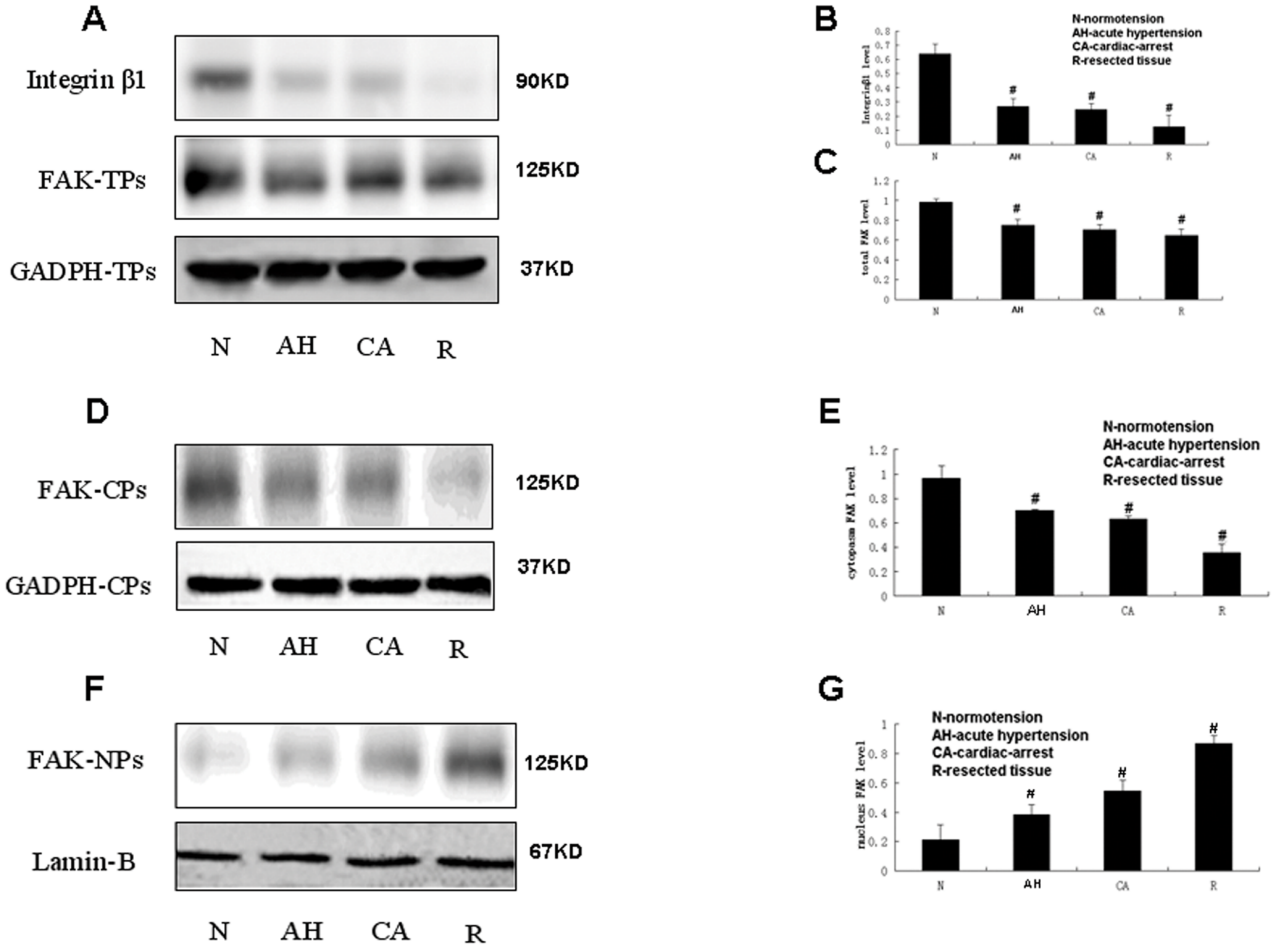
The effects of various hemodynamic conditions on integrin β1 and FAK expression in glomeruli. Representative western blot showing the expression of integrin β1 and total proteins of FAK (FAK-TPs) (A), cytoplasmic proteins (FAK-CPs) (D), and nuclear proteins (FAK-NPs) (F). Quantitative analysis of integrin β1 (B), the total proteins expression of FAK (C), the cytoplasmic proteins expression of FAK (E), the nuclear proteins expression of FAK (G). N: normotension; AH: acute hypertension; CA: cardiac arrest; R: resected tissue. The results are expressed as the OD values of integrin β1 and FAK (means ±SDs); #: P<0.05.

To further evaluate the expressions of integrin β1 and FAK quantitatively, we examined the two podocyte protein levels in glomeruli. From Fig. 5A, we can see two bands at 90 kDa and 125 kDa. Evidently, the band at 90 kDa represents integrin β1,and the other band corresponds to FAK. The total protein levels of integrin β1 and FAK were consistently decreased under abnormal hemodynamic conditions compared to normotensive condition. To further confirm the nuclear translocation of FAK under abnormal conditions, we extracted the nuclear proteins from glomeruli and performed western blot. We found that FAK in cytoplasmic protein was gradually reduced, but it apparently increased in nuclear protein. These results suggested that FAK was translocated to the nuclei under abnormal hemodynamic conditions.

All of the results were consistent with the immunostaining results and could be attributed to the alteration of renal hemodynamic conditions.

## Discussion

It is well known that podocytes are the targets of many forms of injury, and hypertensive injury might be one of the major causes, however, the precise mechanism has not been fully elucidated. In the present study, we visualized the alteration and rearrangement of integrin β1 and FAK under different hemodynamic conditions and as evidenced by IVCT with immunohistochemistry, immunofluorescence and western blot analysis. The results exhibited the reduction and transposition of integrin β1 and FAK under abnormal hemodynamic conditions.

It has been generally accepted that kidney morphology and function are dependent on the maintenance of normal blood pressure [10]. Additionally, hemodynamic factors change instantaneously [20]. To study these changes accurately, we must identify a method that can capture all of the biological components in situ. IVCT has been confirmed as having these capabilities [10], [20]. This technique overcomes the technical problems of conventional fixation processes by cryofixing target organs in situ under living status, which could clarify the native morphological features of kidney tissues. In addition, IVCT can capture more molecular translocation events instantly in situ, which is much closer to the living state. Meanwhile, we can detect the erythrocytes in capillary vessels. We stained the nuclear with DAPI, ensuring the expression changes of integrin β1 and FAK in podocytes, excluding the expression of them in erythrocytes (Fig S1).

Under normotensive condition, integrin β1 was continuous and linear-like along the glomerular capillary loop, which is consistent with the previous report [21]. Reduced expression of integrin α3β1 in podocytes has been demonstrated in humans with FSGS [22], in diabetic nephropathy [5], and in PAN model rats [23]. However, how it changes under abnormal hemodynamic conditions in vivo has not been studied to date. Our study revealed the downregulation of integrin β1 in the prophase of acute hypertension, cardiac arrest. Furthermore, we found integrin β1 attached to the basal membrane of the podocytes, and the foot processes appeared with different degrees of fusion under hypertensive condition, which might strengthen the adhesion force between podocytes and GBM, while under normotensive condition, they were distributed in the basolateral membrane. This ultrastructural appearance was similar to a previous study [24]. Consistent with our observation, Cecile Dessapt exposed podocytes to the FX3000 strain unit (Flex-cell Int, USA), which can mimic glomerular hypertension, demonstrating that integrin β1 was downregulated under hypertensive condition [25]. Many studies have reported that integrin β1 downregulation is causally related to the loss of podocytes, which was supported by our recent observations (Wang, Li et al. unpublished data), in which we found podocytes in the urinary sediment of chronic hypertension patients, accompanied by a parallel reduction of podocytes in renal biopsy tissue. Additionally, another study suggested that α-actinin-4, which can regulate integrin β1 activation, is indispensable for maintaining strong podocyte adhesion to GBM [26]. FAK, as a signal molecule, has been demonstrated to be a key factor in mediating cell adhesion, and FAK activation was highly correlated with cell adhesion force [27]. In the present study, FAK and pTyr-397 FAK were visualized in the cytoplasm and nuclei of the podocytes in normotensive group, whereas the total expression of the two decreased under hypertensive condition. In contrast, the previous study suggested that pTyr397-FAK increased under pathological conditions, such as low-dosage LPS injections and rabbit anti-GBM-induced podocyte injury [7]. One possible explanation for these paradoxical findings is that abnormal hemodynamic injury is underlain by a different mechanism than other injuries. Another possible explanation for the discrepancy is the observation time. We observed the change immediately after ligating the aorta, which could be realized by IVCT. In comparison, they were observed several hours after treatment. Herein, we can speculate that the FAK and pTyr397-FAK experience a transient reduction during the prophase of podocyte injury. However, the precise underlying mechanisms of adhesion are not yet clear. A previous study suggested that FAK up-regulated integrin activation to enhance integrin binding [28]. Conversely, another study reported that FAK was the downstream reactor with integrin, augmenting osteoblast adhesion [29]. However, which is the upstream of the signalling pathway under abnormal hemodynamic conditions has been a matter of debate until now.

Additionally, ischemia and hypoxia are important factors for podocytes. Several studies have documented that ischemia and hypoxia are widely associated with podocytes, except for in tubular and endothelial damage. Mark C. Wagner found that podocytes became swollen, accompanied by narrowed filtration slits, after stopping blood flow for 45 minutes [30]. Similarly, another report showed that podocytes were flattened and that processes widened after ischemia [31]. Furthermore, podocytes were demonstrated to be susceptible to apoptosis after exposure to low oxygen [32]. However, the molecular events that induce podocyte injury during ischemia are unknown. In this study, it was shown that the total proteins levels of integrin β1 and FAK decreased under cardiac arrest conditions compared to normotensive condition. In comparison, the level of FAK in the nuclei increased. Thus, we speculate that the accumulation of FAK in the nuclei is likely to participate in the regulation of ishchemic injury. In the immersion fixation group, the expression of integrin β1 and FAK was theoretically the least in all of the groups, because that group underwent the longest duration of ischemia. Unexpectedly, the expression of integrin β1 appeared the highest by immunohistochemistry. A possible explanation is that many superficial proteins of integrin β1 were redistributed or even washed off during the immersion fixation steps. Thus, what we observed was unture. This conclusion could be further confirmed by western blot analysis.

In summary, we visualized the alteration of integrin β1 and FAK during the prophase of the abnormal hymodynamic conditions using IVCT. Integrin β1 and FAK are two essential factors in the regulation of podocyte changes under abnormal hemodynamic conditions. Although the detailed molecular mechanism is not yet clear, pTyr397-FAK is absolutely necessary for the regulation of podocyte changes. Above all, IVCT can maintain all of the substances in situ, providing accurate and clear images. Our next step is to investigate the associated molecules in the integrin-FAK signaling pathway, which should provide new insights into the regulation mechanisms during hemodynamic disorders.

## Supporting Information

Figure S1 Confocal laser scanning micrographs showing the co-localization of integrin β1 and FAK on podocytes. In normotensive condition, the micrographs of (A–D) show the localization of integrin β1 (A), FAK (B), and the co-localization of integrin β1 and FAK (D). E, F, G and H present the control stainings ommitting the primary antibodies. The micrographs of (I–L) show the localization of them in hypertensive condition, while (M–P) show the control stainings ommitting the primary antibodies. In the (C, G, K, O), we detect the pococyte nuclei staining with DAPI. After merging, the overlapping images of Integrinβ1 and FAK was detected, represented by purple color (arrows).
(TIF)

## References

[1] Miner JH (2011) Glomerular basement membrane composition and the filtration barrier. Pediatr. Nephrol 26: 1413–1417. 10.1007/s00467-011-1785-1 PMC3108006 21327778

[2] Guerrot D, Dussaule JC, Mael-Ainin M, Xu-Dubois YC, Rondeau E, et al (2012) Identification of periostin as a critical marker of progression/reversal of hypertensive nephropathy. Plos One 7: e31974. 22403621 10.1371/journal.pone.0031974 PMC3293874

[3] Kretzler M, Koeppen-Hagemann I, Kriz W (1994) Podocyte damage is a critical step in the development of glomerulosclerosis in the uninephrectomised - desoxycorticosterone hypertensive rat. Virchows Arch 425: 181–193. 7952502 10.1007/BF00230355

[4] Wang G, Lai FM, Kwan BC, Lai KB, Chow KM, et al (2009) Podocyte loss in human hypertensive nephrosclerosis. Am J Hypertens 22: 300–306. 19131934 10.1038/ajh.2008.360

[5] Chen HC, Chen CA, Guh JY, Chang JM, Shin SJ, et al (2000) Altering expression of α3β1 integrin on podocytes of human and rats with diabetes. Life Sciences 67: 2345–2353. 11065181 10.1016/s0024-3205(00)00815-8

[6] Mitra SK, Hanson DA, Schlaepfer DD (2005) Focal adhesion Kinase: In command and control of cell motility. Molecular Cell Biology 6: 56–68. 15688067 10.1038/nrm1549

[7] Ma H, Togawa A, Soda K, Zhang J, Lee S, et al (2010) Inhibition of podocyte FAK protects against proteinuria and foot process effacement. J Am Soc Nephrol 21: 1145–1156. 20522532 10.1681/ASN.2009090991 PMC3152231

[8] Li ZY, Yi XP, Zhong L, Fa QL, Zhou WY, et al (2007) Expression of focal adhesion kinase in cardiac myocytes of hypertrophic ventricle. Chin J Pathol 36: 677–680. 18194601

[9] Yi XP, Wang X, Gerdes AM, Li F (2003) Subcellular redistribution of Focal Adhesion Kinase and its related nonkinase in hypertrophic myocardium. Hypertension 41: 1317–1323. 12732587 10.1161/01.HYP.0000072772.74183.5F

[10] Ohno S, Terada N, Fujii Y, Ueda H, Takagama I (1996) Dynamic structure of glomerular capillary loop as revealed by an “in vivo cryotechnique”. Virchows Arch 427: 519–527. 8624582 10.1007/BF00199513

[11] Ofstad J, Iversen BM (2005) Glomerular and tubular damage in normotensive and hypertensive rats. Am J Physiol Renal Physiol 288: F665–672. 15536168 10.1152/ajprenal.00226.2004

[12] Christiansen RE, Tenstad O, Leh S, Iversen BM (2004) Glomerular charge selectivity is impaired in hypertensive nephropathy. Nephrol Dial Transplant 19: 1083–1091. 14993487 10.1093/ndt/gfh101

[13] Remuzzi G, Bertani T (1998) Pathophysiology of progressive nephropathies. N. Engl. J. Med. 339: 1448–1456. 9811921 10.1056/NEJM199811123392007

[14] Birn H, Fyfe JC, Jacobsen C, Mounier F, Verroust PJ, et al (2000) Cubilin is an albumin binding protein important for renal tubular albumin reabsorption. J Clin Invest 105: 1353–1361. 10811843 10.1172/JCI8862 PMC315466

[15] Griffith LD, Bulger RE, Trum BF (1967) The ultrastructure of the functioning kidney. Lab Invest 16: 220–246. 4164680

[16] Pagtalunan ME, Olson JL, Tilney NL, Meyer TW (1999) Late consequences of acute ischemic injury to a solitary kidney. J Am Soc Nephrol 10: 366–373. 10215337 10.1681/ASN.V102366

[17] Li ZL, Li K, Zhai XY, Wang LN, Ohno N, et al (2013) MRT Letter: Application of novel “in vivo cryotechnique”in living animal kidneys. Microsc Res Tech 76: 113–120. 23132785 10.1002/jemt.22149

[18] Li ZL, Ohno N, Terada N, Zhou DY, Yoshimura A, et al (2006) Application of periodic acid-Schiff fluorescence emission for immunohistochemistry of living mouse renal glomeruli by an “in vivo cryorechnique”. Arch Histol Cytol 69: 147–161. 17031021 10.1679/aohc.69.147

[19] Holthöfer H, Sainio K, Miettinen A (1991) Rat glomerular cells do not express podocytic markers when cultured in vitro. Lab Invest 65: 548–557. 1753704

[20] Li ZL, Terada N, Ohno N, Ohno S (2005) Immunohistochemical analyses on albumin and immunoglobulin in acute hypertensive mouse kidneys by “in vivo cryotechnique”. Histol Histopathol 20: 807–816. 15944930 10.14670/HH-20.807

[21] Zou MS, Yu J, Zhou JH, Nie GM, Ding DS, et al (2010) 1,25-Dihydroxyvitamin D3 ameliorates podocytopenia in rats with adriamycin-induced nephropathy. Intern Med 49: 2677–2686. 21173542 10.2169/internalmedicine.49.4174

[22] Chen CA, Hwang JC, Guh JY, Chang JM, Lai YH, et al (2006) Reduced podocyte expression of alpha3beta1 integrins and podocyte depletion in patients with primary focal segmental glomerulosclerosis and chronic PAN-treated rats. J Lab Clin Med 47: 74–82. 10.1016/j.lab.2005.08.011 16459165

[23] Kojima K, Matsui K, Nagase M (2000) Protection of alpha(3) integrin-mediated podocyte shape by superoxide dismutase in the puromycin aminonucleoside nephrosis rat. Am J Kidney Dis 35: 1175–1185. 10845833 10.1016/s0272-6386(00)70056-4

[24] Baraldi A, Zambruno G, Furci L, Manca V, Vaschieri C, et al (1994) Beta-1 integrins in the normal human glomerular capillary wall: an immunoelectron microscopy study. Nephron 66: 295–301. 7514744 10.1159/000187826

[25] Dessapt C, Baradez MO, Hayward A, Dei Cas A, Thomas SM, et al (2009) Mechanical forces and TGFβ1 reduce podocyte adhesion through α3β1 integrin downregulation. Nephrol Dial Transplant 24: 2645–2655. 19420102 10.1093/ndt/gfp204

[26] Dandapani SV, Sugimoto H, Matthews BD, Kolb RJ, Sinha S, et al (2007) α-Actinin-4 is required for nornal podocyte adhesion. J Biol chem 282: 467–477. 17082197 10.1074/jbc.M605024200

[27] Ay C, Yeh CC, Hsu MC, Hurng HY, Kwok PC, et al (2012) Evaluationg of the correlation between focal adhesion kinase phosphorylation and cell adhesion force using “DEP”technology. Sensors 12: 5951–5965. 22778624 10.3390/s120505951 PMC3386723

[28] Michanel KE, Dumbauld DW, Burns KL, Hanks SK, Garcia AJ (2009) Focal adhesion kinase modulates cell adhesion strengthening via integrin activation. Mol Biol cell 20: 2508–2519. 19297531 10.1091/mbc.E08-01-0076 PMC2675629

[29] Xu JK, Chen HJ, Li XD, Huang ZL, Xu H, et al (2012) Optimal intensity shock wave promotes the adhesion and migration of rat osteoblasts via integrin β1-mediated expression of phosphorylated focal adhesion kinase. J Biol chem 287: 26200–26212. 22654119 10.1074/jbc.M112.349811 PMC3406705

[30] Wagner MC, Rhodes G, Wang E, Pruthi V, Arif E, et al (2008) Ischemic injury to kidney induces glomerular podocyte effacement and dissociation of slit diaphragm proteins Neph1 and ZO-1. J Biol Chem 283: 35579–35589. 18922801 10.1074/jbc.M805507200 PMC2602882

[31] Racusen LC, Prozialeck DH, Solez K (1984) Glomerular epithelial cell changes after ischemia or dehydration. Possible role of angiotensin II. Am J Pathol 114: 157–163. 6691412 PMC1900389

[32] Brukamp K, Jim B, Moeller MJ, Haase VH (2007) Hypoxia and podocyte-specific Vhlh deletion confer risk of glomerular disease. Am J Physiol Renal Physiol 293: F1397–1407. 17609290 10.1152/ajprenal.00133.2007

