# Supplementary figures and images for: Down-Regulation of Integrin β1 and Focal Adhesion Kinase in Renal Glomeruli under Various Hemodynamic Conditions
Source: PLoS One. 2014 Apr 4;9(4):e94212. doi: 10.1371/journal.pone.0094212 (PMC3976409; doi:10.1371/journal.pone.0094212)

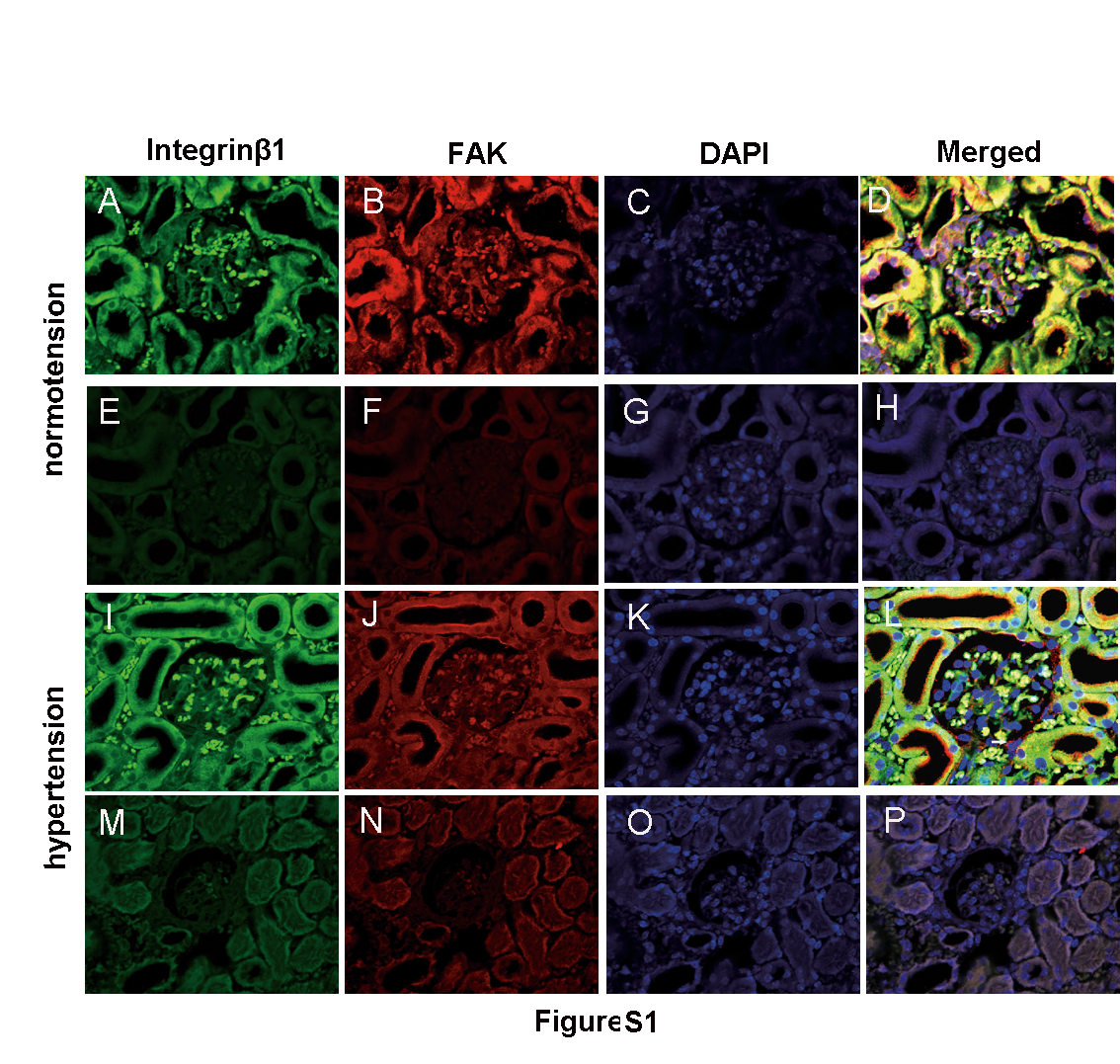

Supplement: Figure S1 — Confocal laser scanning micrographs showing the co-localization of integrin β1 and FAK on podocytes. In normotensive condition, the micrographs of (A–D) show the localization of integrin β1 (A), FAK (B), and the co-localization of integrin β1 and FAK (D). E, F, G and H present the control stainings ommitting the primary antibodies. The micrographs of (I–L) show the localization of them in hypertensive condition, while (M–P) show the control stainings ommitting the primary antibodies. In the (C, G, K, O), we detect the pococyte nuclei staining with DAPI. After merging, the overlapping images of Integrinβ1 and FAK was detected, represented by purple color (arrows). (TIF) [file pone.0094212.s001.tif]
